# A multilevel factor analysis of the short form of the Centrality of Event Scale

**DOI:** 10.3389/fpsyg.2023.1268283

**Published:** 2024-01-05

**Authors:** Daniel Zimprich, Justina Pociūnaitė, Tabea Wolf

**Affiliations:** Department of Developmental Psychology, Institute of Psychology and Education, Ulm University, Ulm, Germany

**Keywords:** Centrality of Event Scale, multilevel factor analysis, positive autobiographical memories, negative autobiographical memories, within persons, between persons

## Abstract

**Introduction:**

The Centrality of Event Scale (CES) has frequently been used to measure the degree to which positive and negative life events are perceived central to a person's identity and life story; and previous research suggests that individuals rate their most positive memory as more central compared to their most negative one. When comparing the centrality of two (or more) memories within individuals, one needs to ensure that the CES (or its short form) is equally valid for different types of events (i.e., positive and negative) as well as on different levels of analyses (i.e., on the between-person and the within-person level), pointing to the issue of measurement invariance.

**Methods:**

Three-hundred sixty-five adults (18–89 years of age) reported up to ten positive and up to ten negative autobiographical memories. For each memory reported, participants completed the seven-item short form of the CES, which measures three different components of centrality: Events can form a central component of identity (two items), a turning point in the life story (three items), and a reference point for everyday inferences (two items).

**Results:**

Based on exploratory and confirmatory factor analyses, we found a two-factor structure (Self-Perception and Life-Course) to fit the data best at both levels of analyses and for both positive and negative events. Strict measurement invariance could be applied for positive and negative events at between-person level and at within-person level. The two factors, which measure the impact of an event on either a person's self-perception or their (future) life course, were rated higher for positive compared to negative memories. This difference, however, was stronger for the self-perception factor.

**Discussion:**

The present study provides a first examination of the factorial structure of the CES short form on two levels (within and between persons) as well as for two types of life events (positive and negative). Whereas, a unidimensional scale might be sufficient to measure the centrality of stressful or traumatic life events, a more fine-graded measure seems better suited to understand the different roles of positive and negative life events for a person's identity and life story.

## 1 Introduction

The Centrality of Event Scale (CES), originally developed to measure whether and, if so, to which degree stressful and traumatic life events have become central to an individual's identity and life story (Berntsen and Rubin, 2006), has extensively been used in research on post-traumatic stress disorder (e.g., Schuettler and Boals, 2011; Groleau et al., 2013), psychopathology (e.g., Pinto-Gouveia and Matos, 2011), and depression (e.g., Newby and Moulds, 2011). Results of these studies have shown that the centrality of a stressful and traumatic event is correlated with the severity of symptoms in post-traumatic stress disorder (Brown et al., 2010), prolonged grief disorder and depression (Boelen, 2012), current feelings of shame (Pinto-Gouveia and Matos, 2011), and poor physical health outcomes (Boals, 2010).

In the present study, our goal was to shed some light on the measurement properties of (the brief version of) the CES (see below). More specifically, we aimed to clarify whether event centrality can be measured comparably for positive and negative events as well as at two levels of data—between persons and within persons.

### 1.1 Factorial structure of the CES

Although the CES has frequently been used, its factorial structure has been investigated only a few times. Originally, the CES was developed to assess three different possible functions that (traumatic) life events may have (Berntsen and Rubin, 2006). The first function entails how a (traumatic) life event has become a reference point, which, from a functional memory perspective, serves as guidance for future behavior, or for learning from one's past experiences (Pillemer, 2009; Rasmussen and Berntsen, 2009). An exemplar item from the CES capturing this function is “This event has become a reference point for the way I understand new experiences.” A second function captures how a (traumatic) life event is seen as a turning point in one's life. From a life narrative perspective, the traumatic event thus functions as closing one chapter and beginning another (e.g., Habermas, 2019). An item from the CES that reflects this function is “If this event had not happened to me, I would be a different person today.” Finally, the third function addresses how an event has become a part of one's personal identity, such that the event is seen as a symbol or theme in one's life. The CES taps this phenomenon through items such as “I automatically see connections and similarities between this event and experiences in my present life.”

In line with these considerations, an exploratory factor analysis of the CES in an undergraduate sample returned three factors with eigenvalues larger than one (Berntsen and Rubin, 2006). However, because there was a drop in the size of eigenvalues from the first compared to the other two eigenvalues, the authors proposed the 20 items of the CES to be unidimensional, that is, to measure *one* underlying latent variable (or factor) of event centrality. Unfortunately, indexes of model fit, factor loadings, or measures of explained variance were not reported, such that the adequacy of a one-factor model compared to a three-factor model cannot be fully evaluated.

By contrast, in a sample of 195 Brazilian undergraduate students, Gauer et al. (2013) found the 20-item CES to be composed of three orthogonal factors, which they found via exploratory factor analysis followed by varimax rotation. Similar to Berntsen and Rubin (2006), there was a drop in eigenvalues from the first eigenvalue on, but the authors nevertheless opted for a three-factor solution. The interpretation of the three factors was in line with the functions proposed theoretically. Specifically, the first factor, on which 10 items showed loadings >0.45, was interpreted as the extent to which the memory of an event has become a reference point for everyday life. The authors interpreted the second factor, on which seven items had loadings >0.45, as the degree the memory of an event has turned into a central component of a person's identity. Finally, the third factor (three loadings >0.45) measured the amount of which an event reflected a turning point in a person's life story.^1^ Note that the factors were chosen to be mutually uncorrelated (i.e., orthogonal), which, in turn, implies that items can show loadings on all three factors. Because the authors decided to only report factor loadings >0.45, the interpretation of the factors is not completely transparent, since it remains unknown whether items significantly loaded on more than one factor and, if so, how strong these cross-loadings were. Moreover, indexes of model fit were not given in the article.

In a sample of 872 Italian adolescents, Ionio et al. (2018) also found a three-factor solution using confirmatory factor analysis, which mapped the factors proposed theoretically by Berntsen and Rubin (2006). The first factor, on which eight items were designated to load (loadings ranging from 0.60 to 0.78), assessed the extent to which an event had become a reference point for expectations and the attribution of meaning to other personal life events. The second factor, composed of seven items with loadings ranging from 0.65 to 0.78, measured the perception of an event as central to one's personal identity. Finally, the third factor (five items with loadings ranging from 0.73 to 0.83) reflected whether an event was perceived as a turning point in one's life story. In addition, the authors tested for measurement invariance across gender and found that factor loadings and intercepts were equal for females and males, implying strong measurement invariance (Meredith and Horn, 2001). Some relevant information is missing in the article, however. For example, the correlations among factors were not reported. In addition, after having established strong measurement invariances across gender, differences in factor parameters (variances, covariances, and means) have, apparently, not been analyzed.

In a study of 1,079 Portuguese adolescents, Vagos et al. (2018) found a three-factor solution as well, which was based on item content and achieved the best model fit. The first factor (“reference point”, on which 7 items loaded) was similar to that of Ionio et al. (2018). Likewise, the second factor (“turning point”, five items) and the third factor (“personal identity”, six items) showed substantial overlap with the Ionio et al. (2018) solution. Notably, however, the authors excluded Items 2 and 11, such that the analyses were based on 18 items. Strong measurement invariance across female and male subsamples was established and a subsequent comparison of factor means showed that females had lower means on factors 2 and 3. The three factors were strongly correlated, ranging from 0.74 between “reference point” and “personal identity” to 0.85 between “reference point” and “turning point”.

In a sample of 263 adults who had experienced at least one traumatic event, Wamser-Nanney (2019) reported that a CES three-factor solution fit the data adequately. However, the three factors were very strongly correlated (*r* = 0.92–0.96), wherefore the author conducted further analyses with a one-factor model—albeit the one-factor solution only showed a marginal fit for the data and represented a significant decrease in fit compared to the three-factor model.

In a recent article, Bruce and Handal (2023) examined the CES factor structure in a sample of MTurk participants recruited online for a survey-based study on self-reported experiences post-trauma and a sample of students exposed to trauma. For the data analysis, from both studies those participants who described their trauma as either bereavement (*N* = 221) or sexual assault (*N* = 97) were selected, resulting in a sample size of 318 persons. In both groups, a two-factor solution emerged from an exploratory factor analysis using varimax rotation. Notwithstanding, in both groups a one-factor solutions was also evaluated, which accounted for 54 and 61% of variance, respectively. Indexes of model fit were not reported.

To summarize previous research on the factorial structure of the CES, it appears as if three factors may be more appropriate to describe the associations among the 20 items—at least in samples of younger adults and predominantly regarding traumatic, stressful or (the most) negative life events. Moreover, the three-factor solutions, with a grain of salt, map to the theoretical structure suggested by Berntsen and Rubin (2006). At the same time, factors are typically strongly correlated, which is why some authors opted for a one-factor solution (e.g., Wamser-Nanney, 2019; Bruce and Handal, 2023). Note, however, that the strong correlations among factors (which imply strong inter-item correlations) may result from the fact that mostly traumatic and most negative events were evaluated by participants—one would expect relatively strong endorsement of all CES items in this case. What complicates a thorough evaluation of previous studies on the factorial structure of the CES is that different analysis approaches have been used (e.g., orthogonal vs. oblique rotation), results stem from samples differing in the severity of the events evaluated using the CES, different language versions of the CES have been employed, and, finally, relevant information is missing in publications.

### 1.2 The CES short form

Berntsen and Rubin (2006) also suggested a brief version of the CES, composed of those seven items that were most strongly correlated with the total score of the original scale. This brief version has also frequently been used in research on the centrality of life events (e.g., Boals, 2010; Rubin et al., 2014). Only a few studies have examined the factorial structure of this short form and, again, these studies focused exclusively on traumatic, respectively negative life events of young adults. Most of them favor a single factor structure as proposed by Berntsen and Rubin (2006).

For instance, in the aforementioned study, Vagos et al. (2018) not only investigated the factorial structure of the full version, but also of the short form of the CES. The authors specified three measurement models, the unidimensional model suggested by Berntsen and Rubin (2006), a unidimensional model based on the seven strongest items correlations suggested by Gauer et al. (2013), and, finally, a three-factor model representing the theoretically postulated components of the CES (i.e., reference point, turning point, and personal identity). The authors favored the unidimensional solution suggested by Gauer et al. (2013), although the three-factor model showed a better fit in terms of RMSEA, CFI, and SRMR. One has to keep in mind, though, that the short form is comprised of seven items only, implying that either one or two factors can be extracted in a meaningful way (based on the requirement of a minimum of three indicators per factor).

Galán et al. (2017) also tested the factorial structure of both the full and short version of the CES in a sample of undergraduates from Spain. Based on two confirmatory factor analyses, their findings support a single factor structure for both CES versions. It is unclear, however, whether other CFA models with more than one factor were tested, because the authors reported results for the single factor solutions only. The same holds for a study conducted by Azadfar et al. (2022). These authors tested the unidimensional structure of the CES short form (and only the single factor structure) in a sample of Iranian university students with a history of at least one romantic breakup, on which the CES measure was based on. Measurement invariance analyses showed that the single factor structure of the CES short form was invariant across gender.

Vermeulen et al. (2020) based their analyses on a sample of 311 Dutch-speaking psychology students (mostly female). Their data favor a single factor solution based on a factor analysis for ordered-categorical data. However, the authors found the best fit for a model that is comprised of six items only (excluding item 7: “This event was a turning point in my life”).

With respect to the CES short form, results of previous studies appear much more unequivocal. In general, a one-factor solution seems to capture the associations among the seven items adequately. However, as for the full CES, relevant information that would help evaluate findings more carefully is lacking in almost all studies.

### 1.3 Centrality of positive and negative events

More recently, the CES (most frequently in its brief version) has also been applied to assess the event centrality of non-traumatic autobiographical events, for example, positive vs. negative life events. Based on the so-called “positivity bias” in autobiographical memory (Walker et al., 2003), individuals are expected to focus on positive information about their personal past more strongly than on negative information. Similarly, the “fading affect bias” (Walker et al., 1997) suggests that the affect intensity of negative events decreases more quickly across time than the affect intensity of positive events (see Hoehne, 2023). The assumption thus is that individuals tend to assign stronger centrality ratings to emotionally positive events compared to emotionally negative events (Pociūnaitė and Zimprich, 2023).

In line with this assumption, Berntsen et al. (2011) found that in older persons the centrality ratings differed in dependence on whether the life event was positive or negative, with the former having a significantly higher event centrality. Similar findings were reported by Zaragoza Scherman et al. (2015). Their study included middle-aged and older adults from Mexico, Greenland, China, and Denmark. Participants completed event centrality scales for their most positive and most negative life events. Across cultures, participants rated positive events as more central than negative events. The same authors conducted a similar study to compare centrality ratings for highly positive and highly negative memories in a sample including young and middle-aged adults, again from Mexico, Greenland, China, and Denmark (Zaragoza Scherman et al., 2020). Both age groups rated their positive memories as more central compared to their negative memories. However, the relative difference between those ratings was smaller in the young adults group (younger adults reported a lower centrality of positive memories than middle-aged adults did). This aligns with studies focusing on samples of younger adults that found no differences in the event centrality ratings between positive and negative events (see Rasmussen and Berntsen, 2009; Boals, 2010, but see Rasmussen and Berntsen, 2013).

Note that one precondition to compare the centrality of emotionally positive vs. emotionally negative autobiographical events is that the CES (or its short form, which was mainly used in previous studies) is equally valid for both types of events. If this precondition does not hold, observed score differences (i.e., CES means of positive vs. negative events) will not accurately reflect true differences in the quantity being measured (i.e., centrality). Psychometricians have developed theory and methods for assessing whether scores are equivalent in meaning and metric across individuals and/or within individuals (e.g., judging the centrality of positive vs. negative events), a condition referred to as measurement invariance (Meredith, 1993; Meredith and Horn, 2001). What we refer to here is not measurement invariance between (groups of) persons—something that has already been examined by Vagos et al. (2018), for example, with respect to males and females. Our concern here is measurement invariance within persons, that is, whether centrality is measured in a comparable manner for positive and negative events when individuals rate centrality for both event types.

### 1.4 The present study: a multilevel perspective on event centrality

In the present study, we approach the measurement of event centrality from two different, but related perspectives, a within-person and a between-person perspective. Moreover, these two perspectives will be adopted for both positive and negative events (cf. Pociūnaitė et al., 2022).

The measurement of event centrality can help answering two conceptually different questions. The first question touches upon the measurement of *differences between persons* in the sense of, for example, examining whether persons with post-traumatic stress disorder symptoms judge the centrality of a stressful event higher than persons with no post-traumatic stress disorder symptomatology. This type of investigation, which can be described as examining between-person or *inter*individual differences in event centrality, is the predominant way the CES has been used in previous studies (e.g., Ionio et al., 2018; Bruce and Handal, 2023).

There is a second perspective on event centrality. If participants are asked, for example, to judge the centrality of events forming their emotionally most positive vs. their emotionally most negative autobiographical memories, the measurement of event centrality can also refer to *within-person* or *intra*individual differences, that is, differences among events. For example, the event centrality of an emotionally negative event might be higher within individuals than that of an emotionally positive event (e.g., Zaragoza Scherman et al., 2015). This within-person perspective comes into play as soon as participants are asked to rate the event centrality of more than one event from their past.

These two types of measuring event centrality—one within-person, the other between-person—can be systematically compared with respect to their measurement qualities by imposing different degrees of measurement invariance (see below). Even more options to examine measurement invariance come into play when the within- and between-person perspectives are transferred to event centrality measurements of positive vs. negative events.

More specifically, in the present study we address the following research questions: (1) Is the measurement of event centrality (as measured by the brief CES) comparable for positive and negative events? (2) Is the measurement of event centrality comparable within and between persons? (3) Combining questions (1) and (2), is the measurement of event centrality comparable both for positive and negative events *and* within and between persons?

## 2 Methods

### 2.1 Sample

The sample of the present study comprised 365 adults aged between 18 and 89 years (*M* = 49.58, *SD* = 17.05).^2^ The majority of the sample was female (67.1%). Participants were mostly married (58.6%) or single (28.8%). Almost half of the sample had graduated from university (45.2%). Sixty-two participants were university students (17%). Most of them belonged to the group of young adults (*n* = 60). The majority of the sample reported to be employed, but occupational status differed considerably with age. Overall, subjective health was rated as good (*M* = 2.23, *SD* = 0.88) on a scale ranging from excellent (1) to poor (5).

Participants were recruited through promotional flyers, e-mail, and word of mouth. To participate in the study, individuals had to be at least 18 old and have a good working knowledge of the German language. After finishing the study, they could take part in a lottery to win a gift voucher (worth 15 Euros). For students, there was an option to get course credit (instead of lottery).

### 2.2 Procedure and measures

Data were collected online using the www.soscisurvey.de platform (Leiner, 2019). After having given their informed consent, participants provided demographic information (e.g., age, gender, marital status, education) and rated their subjective health. Next, participants were asked to recall up to ten positive memories. They were instructed to briefly describe the (first) memory that came to their mind. Participants were told that memories did not have to be extraordinary, but should refer to a specific and distinct event from their personal past. For each memory, a separate page was provided where participants were asked to enter a brief description of the event and proceed to the next memory once they were finished. In the next step, participants were asked to recall up to ten negative memories. The instruction and the procedure were identical to the one for positive memories. If participants did not find 10 positive and/or 10 negative memories to report, they could proceed to the next page. Order of the procedure was the same for all participants.

After having described positive and negative memories, participants completed a personality questionnaire. Subsequently, participants were presented with their description of positive and negative memories and were asked to answer several questions concerning the events described (see below). Memories were presented in the order in which they had been recalled (again, starting with positive and then negative memories).

*Centrality of event*. Participants rated the event centrality for each reported memory. We used the seven-item short version of the CES, which—as suggested by Berntsen and Rubin (2006)—consists of Items 3, 6, 10, 12, 16, 17, and 18 of the original CES. Responses were made on a 5-point Likert-scale ranging from totally disagree (1) to totally agree (5). German item wordings were based on the translation of two independent researchers and are very similar to those of the recently published German version of the full CES (Conen et al., 2022).

### 2.3 Modeling approach

The data in the present study represent a typical multilevel situation, where measurements (centrality of event of different positive and negative autobiographical memories) are nested within persons (Hox, 1995). Consider a multivariate situation of multilevel data, in which there are *i* = 1, …, *N* individuals (Level 2) and within each individual, there are *p* variables (i.e., the seven CES items) measured with respect to *j* = 1, …, *m*_*i*_ autobiographical memories (Level 1).^3^ Let **y**_*ij*_ denote the *p*×1 vector of CES items measured in individual *i* with respect to autobiographical memory *j*. Suppose that this vector of measured variables is composed as


$$\begin{array}{l} {\text{y}}_{ij}=\mu +{\text{v}}_{i}+{\text{w}}_{ij}, \end{array}$$

where **μ** is a *p*×1 vector of overall (or sample) means of the CES items, **v**_*i*_ is a *p*×1 vector of deviations of the individual-specific means of the CES items from the overall means (i.e., ${\text{v}}_{i}={\bar{\text{y}}}_{i}-\mu$, where ${\bar{\text{y}}}_{i}$ denotes the vector of individual-specific means of the CES items) and **w**_*ij*_ is a *p*×1 vector of memory-specific deviations from the individual-specific mean deviations (i.e., ${\text{w}}_{ij}={\text{y}}_{ij}-{\bar{\text{y}}}_{i}$). The vectors **v**_*i*_ and **w**_*ij*_ are independent with expectations $E\left({\text{v}}_{i}\right)=E\left({\text{w}}_{ij}\right)=\text{0}$ and covariance matrices $C\left({\text{v}}_{i}\right)=\Sigma_{2}$, the covariance matrix of *inter*individual (or between-person) differences, and $C\left({\text{w}}_{ij}\right)=\Sigma_{1}$, the covariance matrix of *intra*individual (or within-person) differences. Assume that the between-person or interindividual differences at Level 2 can be described by a factor analysis model (Longford and Muthén, 1992) such that


$$\begin{array}{l} {\text{v}}_{i}=\Lambda_{b}\xi_{i}+{\text{u}}_{i}, \end{array}$$

where **Λ**_*b*_ is a *p*×*q* matrix of factor loadings at Level 2 (or the between-person level), **ξ**_*i*_ is a *q*×1 vector of factor scores of individual *i* at Level 2, and **u**_*i*_ is a *p*×1 vector of residuals at Level 2. Factor scores are assumed to be normally distributed with zero means and covariance matrix **Φ**, that is, $\xi_{i}\sim N\left(\text{0},\Phi \right)$. Similarly, residuals are normally distributed with zero means and covariance matrix **Θ**_*u*_, that is, ${\text{u}}_{i}\sim N\left(\text{0},\Theta_{u}\right)$. Assuming that factor scores and residuals are independent, the between-person or Level 2 covariance matrix predicted by the factor analysis model is


$$\begin{array}{l} \Sigma_{2}={\text{v}}_{i}{\text{v}}_{i}^{\prime }=\Lambda_{b}\Phi \Lambda_{b}^{\prime }+\Theta_{u}. \end{array}$$

Moreover, suppose that the within-person or intraindividual differences can also be described by a factor analysis model, that is,


$$\begin{array}{l} {\text{w}}_{ij}=\Lambda_{w}\eta_{ij}+{\text{e}}_{ij}, \end{array}$$

where **Λ**_*w*_ is a *p*×*r* matrix of factor loadings at Level 1 (or the within-person level), **η**_*ij*_ is a *r*×1 vector of factor scores at Level 1, and **e**_*ij*_ is a *p*×1 vector of residuals at Level 1. Both factor scores and residuals at Level are assumed to be independent and normally distributed with zero means and covariance matrices **Ψ** and **Θ**_*e*_, respectively, that is, $\eta_{ij}\sim N\left(\text{0},\text{Ψ}\right)$ and ${\text{e}}_{ij}\sim N\left(\text{0},\Theta_{e}\right)$. The predicted Level 1 covariance matrix then is


$$\begin{array}{l} \Sigma_{1}={\text{w}}_{ij}{\text{w}}_{ij}^{\prime }=\Lambda_{w}\text{Ψ}\Lambda_{w}^{\prime }+\Theta_{e}. \end{array}$$

The total covariance matrix of observed variables is thus equal to (cf. McDonald, 1993)


(1) $$\Sigma_{\text{total}}=\Sigma_{2}+\Sigma_{1}=\underset{\text{between-person}}{\underset{︸}{\Lambda_{b}\Phi \Lambda_{b}^{\prime }+\Theta_{u}}}+\underset{\text{within-person}}{\underset{︸}{\Lambda_{w}\Psi \Lambda_{w}^{\prime }+\Theta_{e}}}.$$

The Level 2 or between-person part of Equation (1) is to be interpreted in line with conventional factor analysis, that is, the between-factors and the between-residuals refer to *inter*individual differences. The within part in Equation (1), however, differs from standard factor analysis in that it reflects the associations among *intra*individual differences (cf. Mehta and Neale, 2005). Here, factors capture shared within-person differences in judging the event centrality of different autobiographical memories.

### 2.4 Multilevel measurement invariance

Measurement invariance (MI) in general—and in a multilevel situation in particular—is a matter of degree (e.g., Zimprich et al., 2005, 2006, 2012; Zimprich and Martin, 2009; Wolf and Zimprich, 2015). More specifically, one may distinguish four forms of measurement invariance (cf. Meredith, 1993; Meredith and Horn, 2001). *Configural invariance* entails that the number of factors and the according salient and non-salient loadings are equal at both levels, i.e., within and between persons, which ensures that the dimensionality of the measured construct is equivalent. *Weak invariance* (or pattern invariance) requires that factor loading matrices be fully invariant within and between persons, i.e., **Λ**_*w*_ = **Λ**_*b*_. On a conceptual level, weak invariance ensures that the same manifest variables (the seven CES items) relate to concepts (factors) in the same way. With weak MI holding, factor variances and covariances can be compared across levels, because the factors are scaled equally. *Strong invariance* (or metric invariance) requires that, in addition to factor loading matrices, latent intercepts of the manifest indicators be invariant. As such, because latent intercepts are only estimated at Level 2, it has no direct equivalent in a multilevel factor analysis. Finally, *strict invariance* adds the constraint of residual variances be invariant at both levels. Although, technically, it is possible to impose strict MI (more specifically, equal residual variances) in a multilevel factor analysis, one would typically not expect it to hold because on Level 2 residual covariances are typically much smaller because they represent “average” residual variances across Level 1 units.

As noted above, in the present study centrality of event was rated for up to 10 positive and up to 10 negative events. Comparing the measurement of event centrality across positive and negative events allows for more invariance analyses than by a typical multilevel factor analysis alone. If one combines the two-level data situation with the fact that centrality ratings were given for positive and negative events, a scheme of four (sub-)models emerges that can be examined with respect to their measurement properties. This scheme is shown in Table 1 with an obvious extension of notation using *p* for positive events and *n* for negative events.

**Table 1:** Four models of the CES estimated simultaneously.

	**Positive (** *p* **) events**	**Negative (** *n* **) events**
Level 1: within (*w*) persons	$\Sigma_{1p}=\Lambda_{wp}{\text{Ψ}}_{p}\Lambda_{wp}^{\prime }+\Theta_{ep}$	$\Sigma_{1n}=\Lambda_{wn}{\text{Ψ}}_{n}\Lambda_{wn}^{\prime }+\Theta_{en}$
Level 2: between (*b*) persons	$\Sigma_{2p}=\Lambda_{bp}\Phi_{p}\Lambda_{bp}^{\prime }+\Theta_{up}$	$\Sigma_{2n}=\Lambda_{bn}\Phi_{n}\Lambda_{bn}^{\prime }+\Theta_{un}$
Total covariance structure	**Σ**_*p*_ = **Σ**_1*p*_+**Σ**_2*p*_	**Σ**_*n*_ = **Σ**_1*n*_+**Σ**_2*n*_
Level 2: mean structure	**μ**_*p*_ = **υ**_*p*_+**Λ**_*bp*_**κ**_*p*_	**μ**_*n*_ = **υ**_*n*_+**Λ**_*bn*_**κ**_*n*_

Given this scheme, configural invariance can be investigated for (1) the measurement of event centrality positive and negative events, (2) for the measurement within and between persons, and (3) for positive and negative events *and* for both analysis levels. For weak invariance, the same three types of models can be examined, such that weak invariance can hold across levels, across positive and negative events, and both.

Regarding strong invariance—which cannot be tested across levels—we can investigate the equality of item intercepts across positive and negative events on Level 2 (between persons). This requires additional notation as shown in Table 1 under “mean structure.” Here, **μ**_*p*_ and **μ**_*n*_ are the observed means for the CES items as rated for positive and negative events, **υ**_*p*_ and **υ**_*n*_ are the latent intercepts of the CES items for positive and negative events, and **κ**_*p*_ and **κ**_*n*_ are the factor means for positive and negative events. Strong invariance across positive and negative events then implies


$$\begin{array}{l} \upsilon_{p}=\upsilon_{n}=\upsilon . \end{array}$$

Given that weak invariance also holds across positive and negative events, i.e., **Λ**_*bp*_ = **Λ**_*bn*_ = **Λ**_*b*_, we have that


$$\begin{array}{l} \mu_{p}-\mu_{n}=(\upsilon_{p}+\Lambda_{bp}\kappa_{p})-(\upsilon_{n}+\Lambda_{bn}\kappa_{n})\\ \;=\Lambda_{b}(\kappa_{p}-\kappa_{n}), \end{array}$$

which shows that, between persons, factor means can directly be compared across positive and negative events. One has to keep in mind that Level 2 is the only data level where factor mean comparisons appear meaningful, because on Level 1 a comparison of factor means of positive and negative events, if possible, would entail a comparison of 10 positive with 10 negative events or 45 comparisons in total.

### 2.5 Assessing model fit

Typically, the fit of an entire multilevel model is evaluated simultaneously—as it is done in ordinary confirmatory factor analysis, for example (e.g., Zimprich et al., 2005). In multilevel data, however, the sample size is usually much larger at Level 1 (within persons) compared to Level 2 (between persons). In our case, there were 5,081 events reported by 365 individuals. For this reason, the fit of the entire model is likely to be dominated by the (lack of) fit on Level 1 and may not be sensitive enough to model misspecifications at Level 2 (Yuan and Bentler, 2007; Ryu and West, 2009). To overcome this problem of standard fit indexes, two approaches have been developed to evaluate model fit in multilevel structural equation models. One approach utilizes partially saturated models to obtain the fit of the Level 1 and Level 2 models separately (Ryu and West, 2009). The other approach, in a first step, estimates the (asymptotic) covariance matrices of the manifest variables at Level 1 and Level 2, which are then used as input data in single-level structural equation models (Yuan and Bentler, 2007). Unfortunately, neither one of the two approaches can be used when parameters are constrained across levels—as is the case in the present study.

As an alternative, the Standardized Root Mean Square Residual (SRMR) can be calculated for both levels, which is equal to the square root of the squared standardized residual variances and covariances. The SRMR is computed as


$$\begin{array}{l} \text{SRMR}=\sqrt{\frac{1}{t+p}\left(\underset{k\leq l}{\sum }{\left({\hat{\varepsilon }}_{kl}^{*}\right)}^{2}+\underset{k}{\sum }{\left({\hat{\varepsilon }}_{l}^{*}\right)}^{2}\right)}, \end{array}$$

with


$$\begin{array}{l} {\hat{\varepsilon }}_{kl}^{*}=\frac{s_{kl}}{\sqrt{s_{k}^{2}s_{l}^{2}}}-\frac{{\hat{\sigma }}_{kl}}{\sqrt{{\hat{\sigma }}_{k}^{2}{\hat{\sigma }}_{l}^{2}}}\text{ and }{\hat{\varepsilon }}_{k}^{*}=\frac{m_{k}}{\sqrt{s_{k}^{2}}}-\frac{{\hat{\mu }}_{k}}{\sqrt{{\hat{\sigma }}_{k}^{2}}}, \end{array}$$

where $t=\frac{p\left(p+1\right)}{2}$ is the number of (non-redundant) variances and covariances, *s*_*kl*_ denotes the sample covariance between variables *k* and *l*, $s_{k}^{2}$ the sample variance of variable *k*, and $s_{l}^{2}$ the sample variance of variable *l*. The model implied counterparts are ${\hat{\sigma }}_{kl}$, ${\hat{\sigma }}_{k}^{2}$, and ${\hat{\sigma }}_{l}^{2}$. Moreover, *m*_*k*_ and μ_*k*_ denote the sample and model implied mean of variable *k*. The SRMR is suitable for assessing how well the model in question reproduces the observed associations among the variables in an interpretable manner. With a grain of salt, it can be interpreted as the average of the absolute value of residual correlations. The SRMR can be calculated at both Level 1 and Level 2, thus offering a means to evaluate model fit within persons and between persons.^4^ For the SRMR, a cut-off criterium of 0.08 has been recommended as based on simulation studies (Hu and Bentler, 1999).

All analyses reported below were conducted using Mplus, Version 7.11 Muthén and Muthén (2013). The absolute goodness-of-fit of models was evaluated using the Satorra-Bentler corrected χ^2^-test and the Root Mean Square Error of Approximation (RMSEA). In addition, we report the Standardized Root Mean Square Residual (SRMR) for both the within- and the between-person covariance matrix. For both the RMSEA and the SRMR values < 0.08 indicate acceptable model fit, whereas values < 0.06 indicate good model fit (Hu and Bentler, 1999). In comparing the relative fit of nested models, we also detail the Satorra-Bentler corrected χ^2^- difference test (Satorra and Bentler, 2010)—which, however, is expected to show excessive statistical power due to the large sample size. Thus, we based our decisions on which model to accept mainly on the SRMR within and between persons.

One additional remark appears in order here. While on Level 2 (between persons), the seven CES Items for the positive and negative events can covary, this is impossible at Level 1 (within persons), because an event is either positive or negative. As a consequence, while on Level 2 there are $\frac{14\times 13}{2}=91$ covariances, on Level 1 there are only $2\times \frac{7\times 6}{2}=42$ covariances. To make such a model amenable for parameter estimation using MPLUS, the Level 1 covariance between the seven CES Items for the positive events and the seven CES Items for the negative events were constrained to be zero. At the same time, the total number of degrees of freedom was reduced by 49 in each model in order to achieve correct Satorra-Bentler corrected χ^2^-tests and RMSEAs.

## 3 Results

Table 2 contains sample statistics for the seven CES items separately for positive and negative events. Shown are the sample means, within-person (Level 1) standard deviations, between-person (Level 2) standard deviations, and intraclass correlations. Two observations are key in Table 2 : (1) The intraclass correlations show that, in general, the amount of variance is smaller on Level 2 (between persons) than on Level 1 (within persons). In other words, participants differ more with respect to their CES ratings of the 10 positive and 10 negative events they evaluated than they differ from each other. (2) The intraclass correlations are, on average, lower for positive compared to negative events (0.266 vs. 0.358).^5^

**Table 2:** Descriptive statistics of the CES items.

	**Positive events (n = 2,712)**	**Negative events (n = 2,369)**
**CES-Item**	**Mean**	**SD**_*w*_	**SD**_*b*_	**ICC**	**Mean**	**SD**_*w*_	**SD**_*b*_	**ICC**
1. I feel that this event has become part of my identity.	3.637	1.142	0.682	0.263	3.195	1.118	0.862	0.373
2. This event has become a reference point for the way understand myself and the world.	3.161	1.136	0.759	0.309	3.057	1.093	0.857	0.382
3. I feel that this event has become a central part of my life story.	3.495	1.240	0.697	0.240	3.158	1.185	0.859	0.345
4. This event has colored the way I think and feel about other experiences.	2.964	1.133	0.746	0.303	3.199	1.077	0.834	0.375
5. This event permanently changed my life.	3.219	1.367	0.673	0.195	3.029	1.217	0.841	0.323
6. I often think about the effects this event will have on my future.	2.453	1.169	0.805	0.321	2.444	1.173	0.903	0.372
7. This event was a turning point in my life.	2.938	1.378	0.762	0.234	2.749	1.246	0.881	0.334

SD_*w*_, within-person (level 1) standard deviation; SD_*b*_, between-person (level 2) standard deviation; ICC, intraclass correlation.

### 3.1 Multilevel factor analyses

In a first model (Model 1 in Table 3), a one-factor model of centrality was estimated for both positive and negative events and at both levels of analysis (within and between persons) simultaneously.^6^ As can be seen from the fit indexes listed in Table 3, Model 1 did not fit. An exploratory factor analysis indicated that a two-factor model (with Items 1, 2, 3, 4 loading on one factor and Items 5, 6, 7 loading on a second, correlated factor) described the data adequately.^7^ Thus, in Model 1a, these two factors were specified within persons (Level 1), while between persons we continued with one factor. Although Model 1a represented a large improvement of fit compared to Model 1 (see Table 3), the RMSEA was not fully acceptable. Moreover, the SRMR_*b*_ indicated that data were not described adequately on Level 2. For Model 1b, we “reversed” Model 1a by specifying one factor on Level 1 and two factors on Level 2. Although doing so also improved fit considerably compared to Model 1, the RMSEA was even less acceptable.

**Table 3:** Model fit.

**Model**	$\chi_{SB}^{2}$	***df***	**SC**	$\Delta \chi_{SB}^{2}$	Δ***df***	**RMSEA**	**SRMS**_*w*_	**SRMR**_*b*_
1: 1 factor at both levels	3,949^*^	104	0.793			0.085	0.044	0.101
1a: 2 factors within, 1 factor between	2,367^*^	102	0.785	1,060^*a^	2	0.066	0.028	0.082
1b: 1 factor within, 2 factors between	3,241^*^	99	0.818	1,612^*a^	5	0.079	0.043	0.062
2: 2 factors at both levels	1,962^*^	97	0.799	2,203^*a^	7	0.061	0.028	0.062
2a: 2 factors with residual covariances	454^*^	86	0.756	1,078^*^	11	0.029	0.014	0.039
3: 3 factors at both levels	3,208^*^	84	0.723	747^*a^	20	0.072	0.038	0.076
4: weak invariance I	447^*^	91	0.775	3^*b*^	5	0.028	0.014	0.039
5: weak invariance II	468^*^	96	0.778	21^*^	5	0.028	0.014	0.040
6: weak invariance III	623^*^	101	0.805	103^*^	5	0.031	0.013	0.057
7: strong invariance I	929^*^	106	0.811	270^*^	5	0.039	0.013	0.068
7a: strong invariance II	737^*^	107	0.809	263^*^	1	0.034	0.013	0.058
8: strict invariance I	735^*^	112	0.841	14*	7	0.033	0.014	0.059
9: strict invariance II	739^*^	119	0.859	15*	7	0.032	0.014	0.059
10: strict invariance III	1,958^*^	126	0.913	785^*^	7	0.054	0.015	0.260

*p* < 0.01, ^*a*^compared to Model 1, ^*b*^compared to Model 2a. $\chi_{SB}^{2}$, Satorra-Bentler corrected chi-square; *df* , degrees of freedom; $\Delta \chi_{SB}^{2}$, difference in Satorra-Bentler corrected chi-square values; Δ*df* , difference in degrees of freedom; RMSEA,

Root Mean Square Error of Approximation; SRMS_*w*_, Standardized Root Mean Square Residual within persons (Level 1); SRMS_*b*_, Standardized Root Mean Square Residual between persons (Level 2).

Model 4 = on the within-person level, factor loadings are constrained to be equal for positive and negative events, i.e., **Λ**_*wp*_ = **Λ**_*wn*_ = **Λ**_*w*_.

Model 5 = on the between-person level, factor loadings are constrained to be equal for positive and negative events, i.e., **Λ**_*bp*_ = **Λ**_*bn*_ = **Λ**_*b*_.

Model 6 = on both levels, factor loadings are constrained to be equal for positive and negative events, i.e., **Λ**_*b*_ = **Λ**_*w*_ = **Λ**.

Model 7 = intercepts of CES items for positive and negative events are constrained to be equal, i.e., **υ**_*p*_ = **υ**_*n*_ = **υ**.

Model 7a = equality constraint of equal intercepts for positive and negative events relaxed for Item 4.

Model 8 = on the within-person level, residual variances are constrained to be equal for positive and negative events, i.e., **Θ**_*ep*_ = **Θ**_*en*_ = **Θ**_*e*_.

Model 9 = Model 8 plus, on the between-person level, residual variances are constrained to be equal for positive and negative events, i.e., **Θ**_*up*_ = **Θ**_*un*_ = **Θ**_*u*_.

Model 10 = Model 9 plus, on both levels, residual variances are constrained to be equal for positive and negative events, i.e., **Θ**_*u*_ = **Θ**_*e*_ = **Θ**.

**Figure 1:**
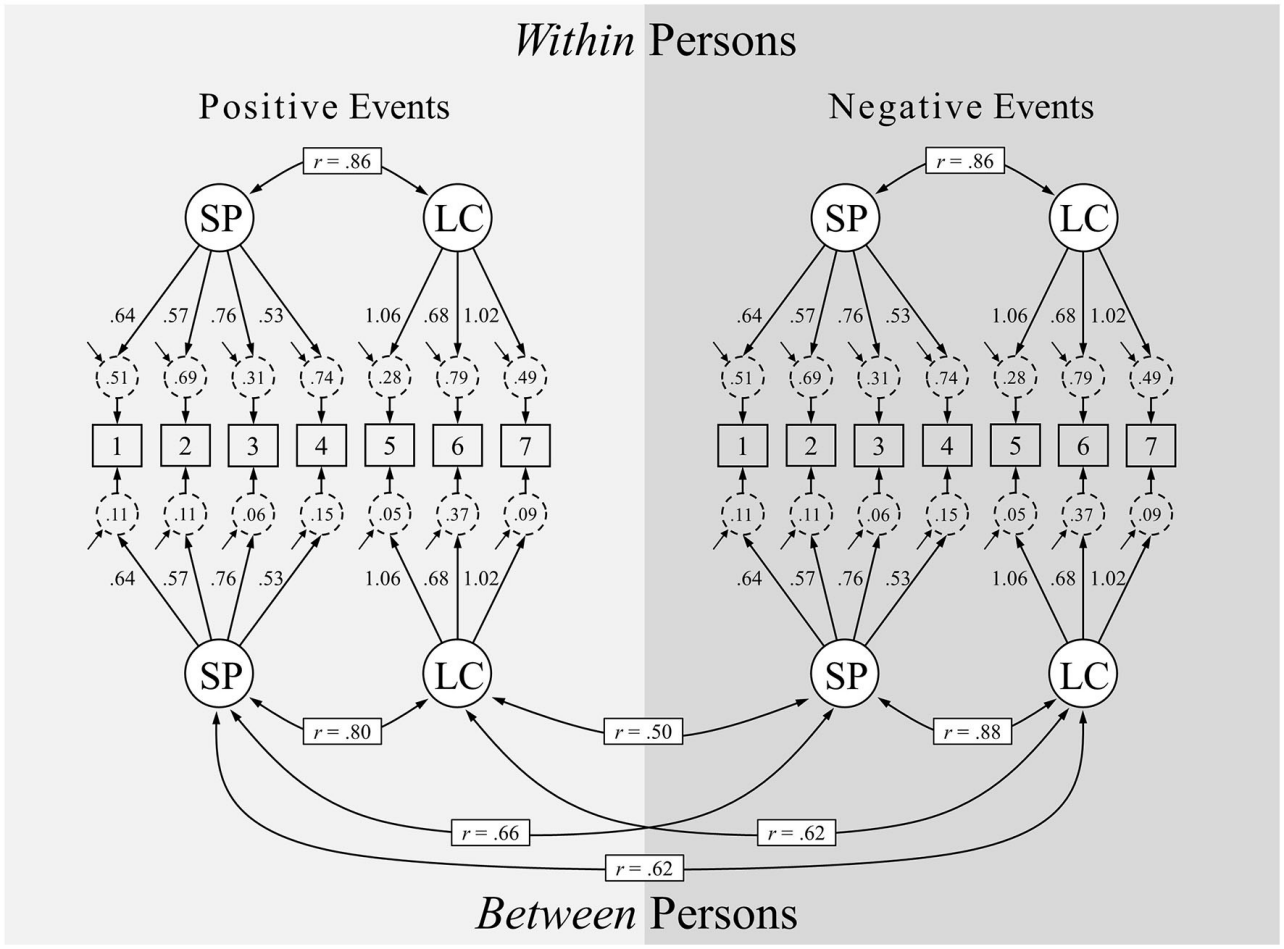
Multilevel factor analysis model of the short version of the Centrality of Event Scale (based on Model 9). SP, impact of an event on a person's Self-Perception; LC, impact of an event on a persons's (future) Life-Course. Apart from inter-factor correlations, parameters are unstandardized.

For Model 2, two factors were estimated at both levels of analysis and for both positive and negative events. This model (see Table 3) showed an acceptable fit. Moreover, it represented a huge improvement of fit compared to Model 1. Based on the factor loadings, we interpreted the first factor as capturing the impact of an event on a person's self-perception—in what follows, we abbreviate this Self-Perception factor as SP. More specifically, the factor captures the amount of which an event became integrated in one's life story and identity. The second factor, by contrast, can be interpreted as the impact of an event on one's (future) life-course—in what follows, we abbreviate this Life-Course factor as LC. Here, the consequences and implications of an event are in focus.^8^ Along another dimension, one could also see the SP factor as capturing the inward-bound impact of an event on the self, requiring integration and reflection, whereas the LC gathers the outward-bound impact of an event on a person's life, being aware of its implications and consequences. Factors were strongly correlated at both levels of analysis and for both positive and negative events (*r*s ranging from 0.52 to 0.88). The model is depicted in Figure 1.

Because according to the RMSEA, fit was at the boundary of the typical cut-off (0.06) of good model fit, in the next model (Model 2a in Table 3), we introduced covariances between the same respective items for positive and negative items on Level 2, the between-person level (i.e., between Item 1 for positive events and Item 1 for negative events, etc.).^9^ Moreover, on Level 1 (within persons), we introduced residual covariances between Items 1 and 2 for positive and negative events and for Items 2 and 4 for positive and negative events.^10^ This model (Model 2a) showed an improved fit, which, in addition, represented an improvement compared to the previous model.

For reasons of completeness, we also estimated a three-factor model with the seven items designated to load on their respective theoretically proposed factors. As can be seen from Table 3, this model (Model 3) did not describe the data well. Furthermore, factors virtually collapsed, that is, their correlations approached unity. Therefore, we decided to continue with Model 2a, which served as the configural invariance model for the measurement invariance analyses.

### 3.2 Measurement invariance analyses

In examining measurement invariance, in a first model (Model 4 in Table 3), we imposed weak invariance with respect to positive and negative events at the within-person level (i.e., **Λ**_*wp*_ = **Λ**_*wn*_ = **Λ**_*w*_). This model showed an acceptable fit, which, moreover, was indistinguishable from that of Model 2a. Based on this result, we concluded that weak MI holds for measuring event centrality for different events (positive vs. negative) within persons.

In the next model (Model 5), the constraint of equal factor loadings for positive and negative events between persons was added (i.e., **Λ**_*bp*_ = **Λ**_*bn*_ = **Λ**_*b*_). Although the Satorra-Bentler corrected χ^2^-difference indicated a significant loss of fit, the RMSEA and both SRMRs remained virtually unchanged, from which we inferred that weak MI holds for measuring event centrality across different events (positive vs. negative) between persons.

Model 6 imposed equal factor loadings across event type and across levels, thus implying “complete” weak measurement invariance (i.e., **Λ**_*w*_ = **Λ**_*p*_ = **Λ**). As Table 3 shows, doing so led to a relatively large decrement in model fit. At the same time, the RMSEA and both SRMRs were still well below their critical cut-off criterium. For this reason, we regarded Model 6 as adequately describing the data.

In Model 7, latent intercepts of the CES items were constrained to be equal across positive and negative events (i.e., **υ**_*p*_ = **υ**_*n*_ = **υ**). From the fit indexes in Table 3, it becomes apparent that this led to a relatively large decrease in fit on Level 2 (in line with the fact that latent intercepts constraints only affect the between-person data level). Upon inspection, Item 4 (“This event has colored the way I think and feel about other experiences.”) showed a large discrepancy for positive and negative events. Therefore, in Model 7a, the constraint of equal intercepts for positive and negative events was relaxed for Item 4. This model showed an almost unchanged fit compared to Model 6. Results showed that Item 4 was endorsed more strongly for negative events than what would have been expected based on the Self-Perception factor differences, while it was endorsed less strongly for positive events. Taken together, only partial strong measurement invariance held across positive vs. negative events.

In Model 8, residual variances were constrained to be equal for corresponding CES items for positive and negative events at the within-person level (**Θ**_*ep*_ = **Θ**_*en*_ = **Θ**_*e*_). As can be seen from Table 3, doing so left model fit almost unchanged. Next, for Model 9, the constraint of equal residual variances for corresponding CES items for positive and negative events at the between-person level was added to Model 8 (**Θ**_*up*_ = **Θ**_*un*_ = **Θ**_*u*_). Again, model fit remained virtually the same. Finally, in Model 10, residual variances were, in addition to Model 9, required to be equal within and between persons (**Θ**_*u*_ = **Θ**_*e*_ = **Θ**). As expected, this model did not achieve an adequate fit.

**Figure 2:**
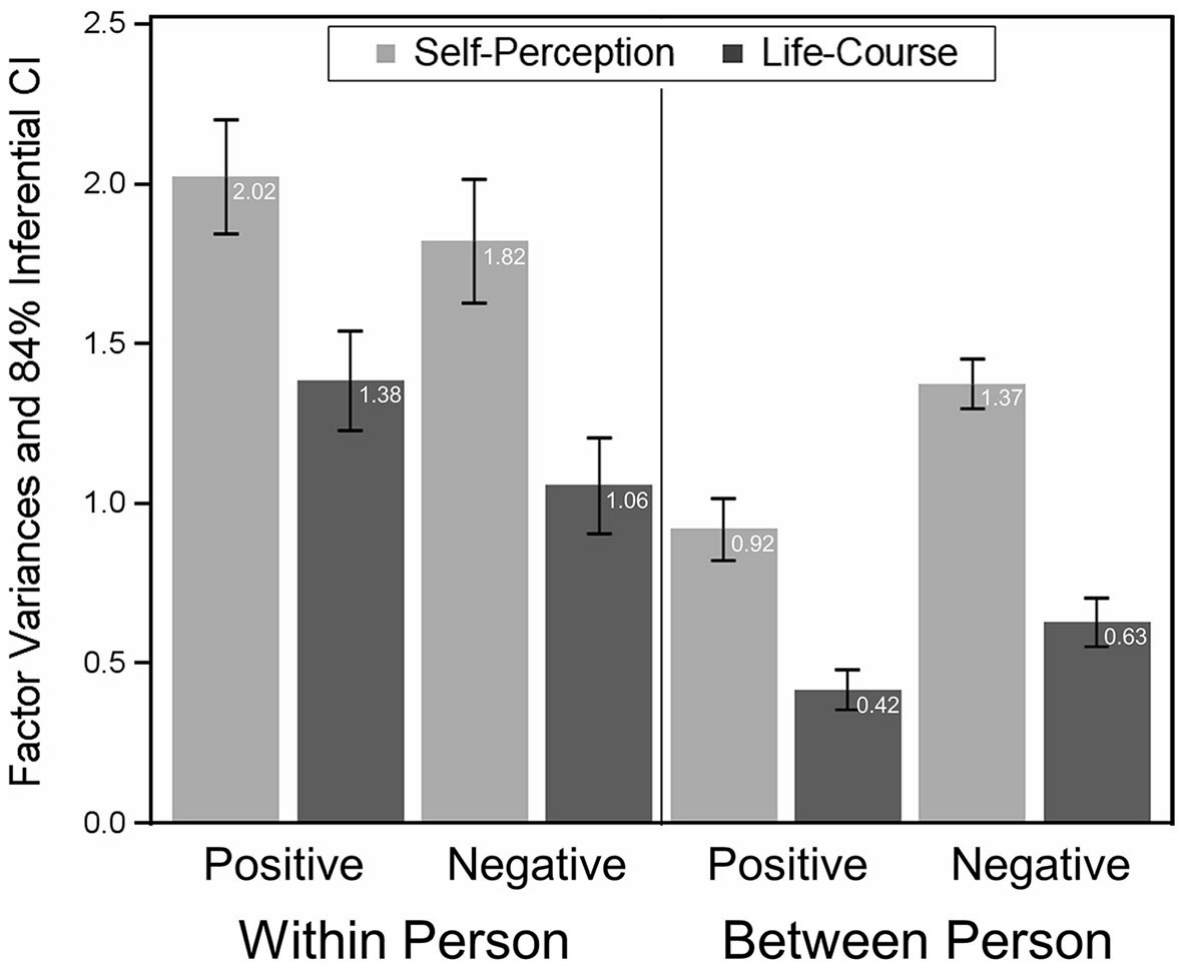
Factor variances of self-perception and life-course for positive and negative events *and* within and between persons. Also shown are the 84% inferential confidence intervals (see Tryon, 2001).

**Figure 3:**
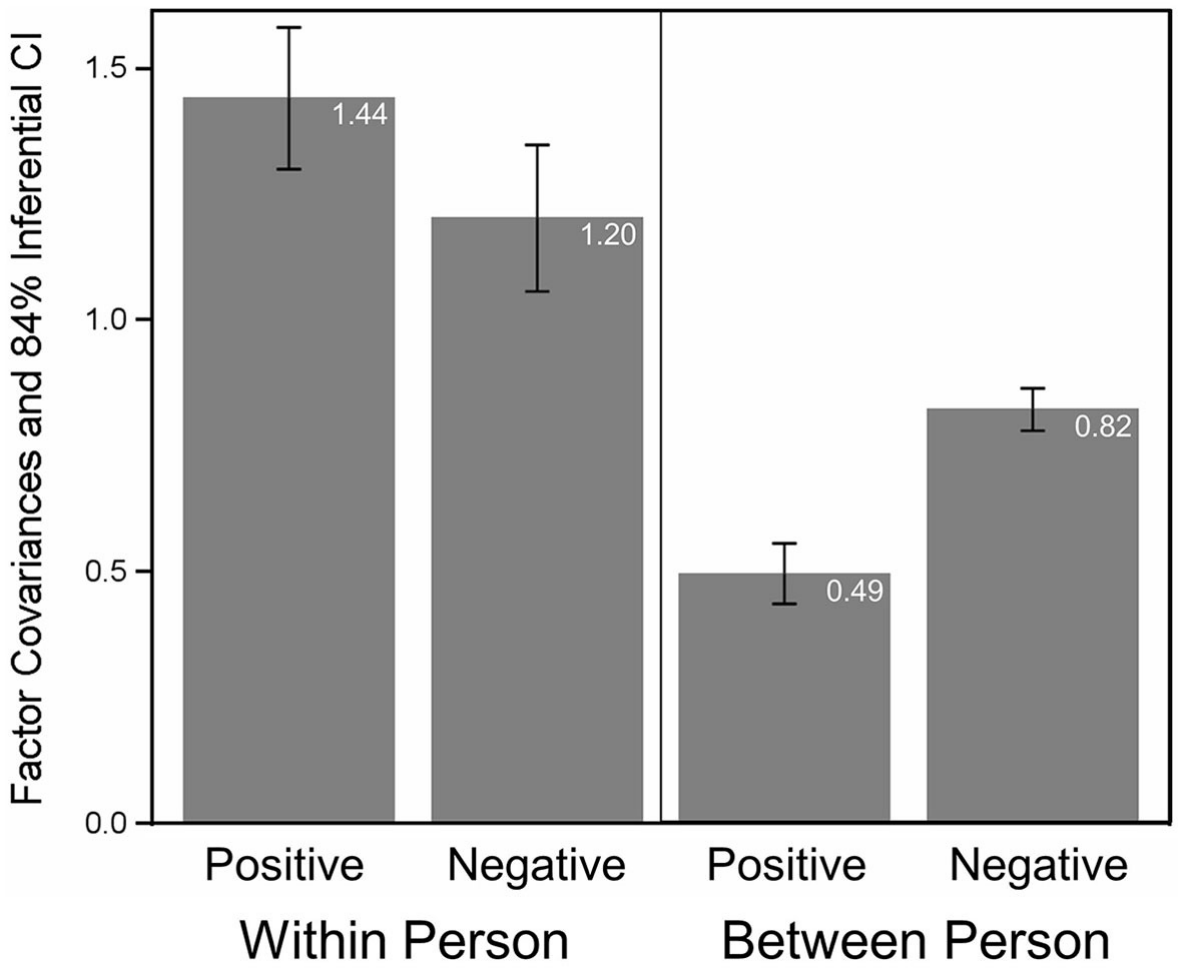
Factor covariances of self-perception and life-course for positive and negative events *and* within and between persons. Also shown are the 84% inferential confidence intervals (see Tryon, 2001).

**Figure 4:**
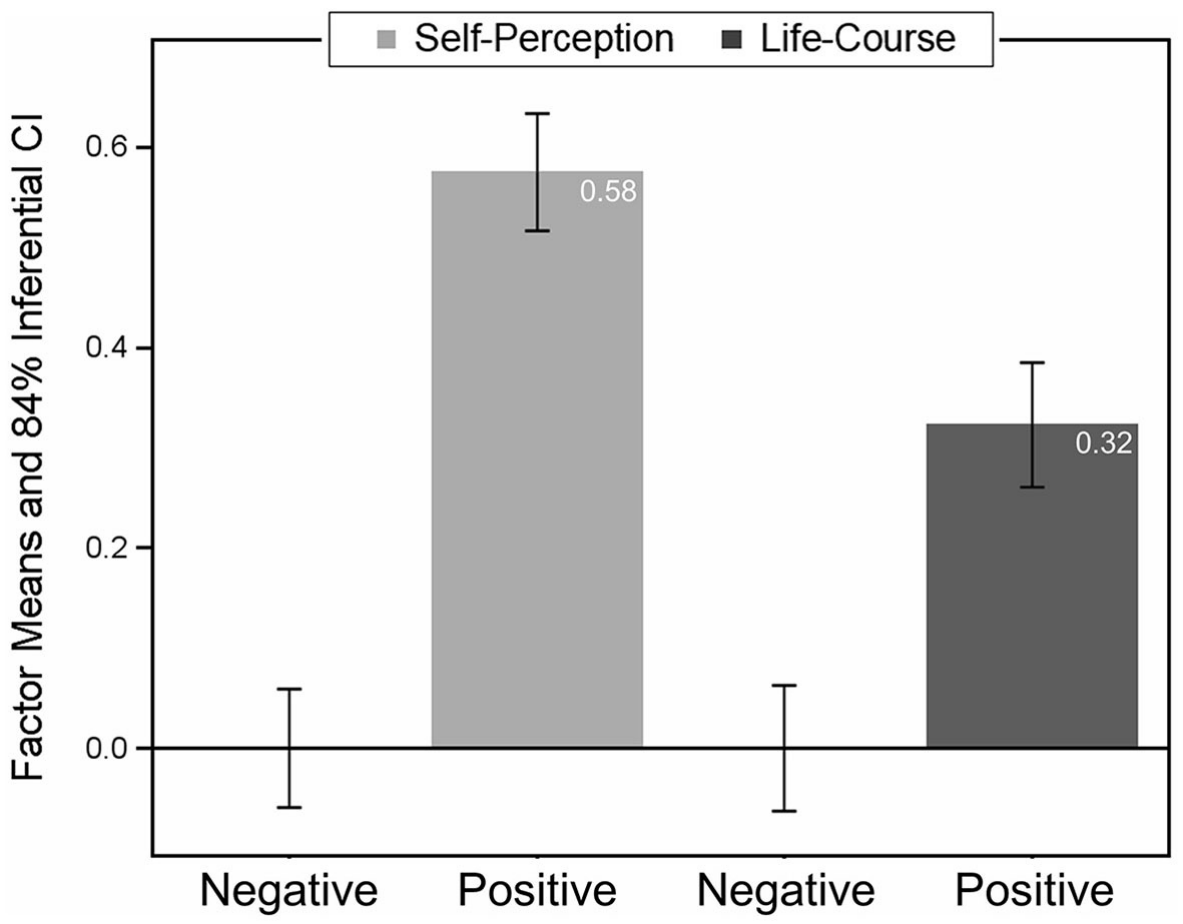
Between-person factor means of self-perception and life-course for positive and negative events (level 2). Also shown are the 84% inferential confidence intervals (see Tryon, 2001).

Summarizing these analyses, we accepted Model 9 as reflecting the associations among CES items for positive and negative events *and* within and between persons adequately. Model 9 entails the following elements of measurement invariance: (1) Factor loadings are completely equal, that is, **Λ**_*wp*_ = **Λ**_*wn*_ = **Λ**_*bp*_ = **Λ**_*bn*_ = **Λ**. This implies that factor variances and covariances can be compared across event types and across data levels. Figure 2 depicts the factor variance estimates based on Model 9. If the 84% inferential confidence intervals (see Tryon, 2001) of (any) two factor variances do not overlap, the variances are significantly different from each other (*p* < 0.05). In line with the descriptive statistics (see Table 2), factor variances were larger on Level 1 (within persons) than on Level 2 (between persons). Moreover, on both levels, the factor variances of Self-Perception were larger than for Life-Course, implying that both event differences and individual differences were more pronounced for Self-Perception than for Life-Course. In addition, Figure 3 shows the factor covariance between Self-Perception and Life-Course for positive and negative AMs and on both data levels. As for the factor variances, factor covariances are much larger on Level 1, implying that the centrality assessments – Self-Perception and Life-Course – are more similar within persons than between persons.^11^ (2) Item intercepts are equal for positive and negative events (except Item 4), implying partial strong invariance. Based on Model 9, factor means can be compared on Level 2 (keeping in mind that factor means were modeled without Item 4).^12^ Figure 4 shows the according factor means scaled in the effect size metric of Cohen's *d*—note that the factor means of the negative events were constrained to be zero for identification purposes, such that the factor means of the positive events represent the difference. The (factor) mean difference of Self-Perception between negative and positive events amounted to an effect size of 0.58, which conventionally would be regarded a medium effect. Thus, the impact of positive events on one's Self-Perception was judged as larger than that of negative events. By contrast, for the difference in Life-Course between negative and positive events, the effect size was 0.32, a small effect. Hence, the impact of positive events on one's Life-Course was larger than that of negative events—although the effect was only about half of the size of the Self-Perception effect. (3) Residual variances of the seven CES items were equal for positive and negative at the within-person and the between-person level, but not across levels. This implies that conditional variance of item responses (given the SP and LC factors), is invariant for intraindividual differences between positive and negative events and interindividual differences.

## 4 Discussion

A person's life story is composed of personally experienced events that are considered highly self-relevant at the time when they took place or which maintain self-importance over time (Bluck and Habermas, 2000; Conway and Holmes, 2004). The life story provides a person with an overall sense of meaningfulness, purpose, and coherence (McAdams, 2001), and thus fosters a sense of self-identity (Conway and Tagini, 2004). However, not all personally experienced events become part of a person's life story; and even those that do, may vary in terms of their self-relevance. For instance, people typically consider their most positive autobiographical memory as more central to their identity than their most negative one (e.g., Zaragoza Scherman et al., 2020; Pociūnaitė and Zimprich, 2023). The centrality of an event may not only vary as a function of valence (i.e., positive vs. negative memories), but also within valence categories in the sense that some positive (or negative) memories contribute strongly to a person's identity and life story, whereas other positive (or negative) events are perceived as less self-relevant. Against this background, it is important to ensure that self-report questionnaires tapping the degree to which autobiographical memories are embedded in a person's life story are reliable measures on both the between-person level as well as the within-person level. The present study provides a first examination of the seven items included in the Centrality of Event Scale (CES) short form. Based on exploratory and confirmatory factor analyses, we found a two-factor structure (Self-Perception and Life Course) at both levels of analyses *and* for positive and negative events.

### 4.1 One or two factors of event centrality?

A few studies have tested the factorial structure of the CES short form and they univocally advocate for a one-factor solution. Given that the short form consists of seven items only, a one-factor solution seems both plausible and practical. Depending on the research question, however, a more fine-graded measure seems warranted; for instance, to understand why some events are perceived as more central than other events. Much like the full version of the CES (e.g., Ionio et al., 2018), the short from is comprised of three, theoretically distinct components: Events can form a central component of personal identity (two items), a turning point in the life story (three items), and a reference point for everyday inferences (two items). These three components are not that clearly mirrored on our two-factor solution. In fact, the first factor included items from all three theoretically postulated factors. What these items share is a focus on the impact of an event on a person's self-perception in the sense that the event shapes how the person thinks and feels about themselves. The first factor, thus, captures the inward-bound impact of an event on the cognitive and emotional level, which requires reflecting on the event and integrating it into one's identity and life story. The second factor consists of items capturing the degree to which an event represents a turning, or reference point; thus only capturing two of the theoretically proposed factors. What these three items share is a focus on the event's impact on a person's life-course—be it in the past or anticipated in the future. Put differently, the second factor describes the outward-bound impact of an event, respectively, the implications and consequences of an event for a person's life.

Notably, these two factors show different patterns in terms of factor variances and covariances on the two data levels: Variances and covariances were much more pronounced within persons than between persons. This implies that individuals differ in assessing the amount of Self-Perception and Life-Course of their individual autobiographical memories (Level 1 variances) but are much more similar when all autobiographical memories are considered together (Level 2 variances). This also shows in the factor covariances, where Self-Perception and Life-Course centrality assessments are more strongly related on Level 1 than on Level 2. In sum, this indicates that there are individuals who tend to go to more extremes in assessing individual positive or negative autobiographical memories, but across all reported autobiographical memories these extremes become more equalized such that individuals are more similar. With respect to factor means (between-person level only), participants generally perceived their positive memories as more central than their negative memories—as indicated by higher factor means for positive compared to negative memories—but this difference was more pronounced for the factor capturing the impact of an event on a person's self-perception (medium effect) compared to the factor describing an event's impact on the life-course (small effect). This implies that both positive and negative events have the potential to change a person's life, be it in a positive or negative way. However, they show distinct contributions to a person's self-perception in the sense that positive events, in particular, shape how a person is thinking and feeling about themselves, their identity, and the world. This aligns with research showing that positive and negative memories serve different functions in daily life (Rasmussen and Berntsen, 2009). For instance, positive memories are more often used to feel better (about oneself), whereas negative memories serve to direct behavior with the goal of avoiding similar experiences, and their negative impact upon one's life in the future (Wolf and Demiray, 2019; Wolf et al., 2021).

Our factorial structure of the short, seven-item version of the CES differs from that found in previous research. Whereas, in previous studies, the short version typically evinced one underlying factor, we found two, albeit substantially correlated factors on the between-person level both for positive and negative events. There are several possible reasons for this discrepancy. First, in our study not traumatic, but simply positive and negative events from their past were assessed using the brief CES. Therefore, one would expect that (a) centrality in our study is, in general, lower than for high-impact, traumatic events and (b) that centrality is more variable across events. This may have led to lower inter-item correlations on both levels of analysis compared to previous studies. Second, between-person differences in our study were not based on having every participant evaluate one event, but result as the individual-specific means across CES items across up to 10 positive and 10 negative events. Associations on Level 2 are expected to be different among items (as analyzed in previous studies) vs. among (latent) person-specific means of items (as analyzed in our study). Importantly, reliability of individual differences can be assumed to be higher in the approach we used (e.g., Muthén, 1991).

To understand the different roles of positive and negative events for a person's identity and life story, a more nuanced centrality measure seems to offer a more fine-graded picture. This does not necessarily imply that a two-factor solution needs to always be applied when using the CES short form. A unidimensional scale might be sufficient when focusing on a person's most stressful or traumatic life event (e.g., Galán et al., 2017; Vagos et al., 2018; Vermeulen et al., 2020; Azadfar et al., 2022), because for highly stressful or traumatic events, one would expect a relatively similar, strong endorsement of all CES items.

### 4.2 Measurement invariance of the brief CES

In the present study—to the best of our knowledge for the first time—the measurement properties of the brief CES were examined both between and within persons and, simultaneously, for positive and negative events (see Figure 1). The model we accepted (Model 9) shows that factor loadings were completely invariant across the quadrants of the scheme in Table 1. That is, weak measurement invariance was established, which allows for a direct comparison of factor variances across event types (positive vs. negative) and across levels of analysis. From Figure 2, it becomes evident that factor variances of Self-Perception and Life-Course were, in general, larger within persons than between persons, implying larger differences among events than among individuals. Moreover, variances of Self-Perception were larger than variances of Life-Course, indicating that the amount of which events become integrated in one's life story and identity (the inward-bound effect of events) was more variable than the amount of which an event has implications and consequences for one's life (the outward-bound effect of events). This appears to suggest that a person can have varying internal interpretations of an event, whereas the external implications is more objective or more universal.

Intercepts of the CES items were not fully invariant across event types because Item 4 showed a pattern different from the remaining items. Whereas, for the other items, both Self-Perception and Life-Course were more pronounced for positive events, amounting to a medium and a small effect (see Figure 4), for Item 4 this pattern was reversed. Thus, negative events appear to color the way individuals think (and feel) about other experiences more than positive events do. This finding has a potentially important consequence: A comparison of the centrality of positive and negative events might better exclude Item 4, because it (with its reverse effect) leads to a downward bias of the event centrality difference. As such, one might suspect that the centrality differences between positive and negative events reported in the literature (e.g., Zaragoza Scherman et al., 2015) may underestimate the true difference.

One implication of weak invariance holding across levels concerns the definition of the intraclass correlation coefficient. Muthén (1991) proposed a “true” intraclass correlation coefficient (ρ_*icc*_), which makes use of the factor-analytic decomposition of the observed variance into a systematic and a residual part and gives the error-free proportion of between-person variance (see Equation 1). For variables that load on one factor only (congeneric model)—as in the present analysis of the brief CES—we have


(2) $$\begin{array}{l} \rho_{icc}=\frac{\lambda_{b}^{2}\phi }{\lambda_{b}^{2}\phi +\lambda_{w}^{2}\psi }, \end{array}$$

where λ_*b*_ is the factor loading of the item in question on Level 2, ϕ is the variance of the factor on Level 2, λ_*w*_ is the factor loading on Level 1, and ψ is the variance of the factor on Level 1. By contrast to the ordinary intraclass correlation coefficient, this “true” intraclass correlation coefficient is not contaminated by measurement error. At the same time, however, it is a model-based quantity based on factor variances, which may take on different values depending on the model used to estimate it. Based on our Model 9 with equal factor loadings on the within-person and the between-person level, Equation (2) can be further simplified, such that (cf. Zimprich and Martin, 2009)


$$\begin{array}{l} \rho_{icc}=\frac{\lambda_{b}^{2}\phi }{\lambda_{b}^{2}\phi +\lambda_{w}^{2}\psi }=\frac{\lambda^{2}\phi }{\lambda^{2}\left(\phi +\psi \right)}=\frac{\phi }{\phi +\psi }, \end{array}$$

implying equal “true” intraclass correlation coefficients for those items loading on the same factor (SP vs. LC). From a substantive perspective equal “true” intraclass correlations appear reasonable: Those variables measuring the same underlying factor have the same ratio of “true” between-person variance in comparison to the total “true” variance—with this ratio being independent of the actual scaling of variables.

## 5 Conclusion

The Centrality of Event Scale (CES) was originally developed to measure the extent of which a traumatic or stressful event becomes integrated into a person's identity and life story. Our findings demonstrate that the CES constitutes a reliable measure to compare the centrality of emotionally positive and emotionally negative memories within and between persons. How the CES is analyzed, however, may depend on the type of events researchers are focusing on. When focusing on traumatic or highly stressful life events, the items of the CES short form may form a single factor. In aiming to understand the different roles of positive and negative events for a person's identity and life story, however, it seems warranted to distinguish between an event's impact on a person's Self-Perception and its consequences for a person's Life-Course. Moreover, the seven items of the CES short form may not be equally suited to meaningfully compare the centrality of positive and negative events (i.e., Item 4). Finally, based on the two-level interpretation, an event can have stronger influences on individual differences in self-perception, whereas the life-course-changing properties of events appear to be less variable across persons.

## Data availability statement

The raw data supporting the conclusions of this article will be made available by the authors, without undue reservation.

## Ethics statement

Ethical approval was not required for the studies involving humans because at the time the study was conducted, ethical approval was not required at Ulm University. The studies were conducted in accordance with the local legislation and institutional requirements. The participants provided their written informed consent to participate in this study.

## Author contributions

DZ: Formal analysis, Investigation, Methodology, Visualization, Writing–original draft, Writing–review & editing. JP: Formal analysis, Methodology, Visualization, Writing–review & editing. TW: Conceptualization, Data curation, Investigation, Project administration, Writing–original draft, Writing–review & editing.

## Footnotes

^1^The amount of explained variance by the respective factors as given in Table 1 in Gauer et al. (2013) was, obviously, calculated before the varimax rotation. Notwithstanding, from these numbers the total sum of variance explained by the three-factor solution can be calculated, which gives 62%.

^2^Part of the data have been used in a previous study with a different focus (see Pociūnaitė et al., 2022).

^3^The subscript *i* for the number *m* of AMs designates that individuals were allowed to differ in the number of AMs they reported.

^4^Note that at Level 1, the part involving means is omitted.

^5^The average within-person correlation among negative events was *r* = 0.549 (Median *r* = 0.535). The average within-person correlation among positive events was *r* = 0.572 (Median *r* = 0.554). Between persons, the average correlation was *r* = 0.615 (Median *r* = 0.606).

^6^On both levels, factors were scaled by fixing the sum of their variances to 1. This scaling appears more adequate than the indicator-variable method, where the loading of one manifest indicator variable is fixed to 1, because in a measurement invariance analysis the latter would already implicitly assume equal factor loadings for this marker variable—something that goes untested until the weak measurement invariance model.

^7^Table A1 in the Appendix contains some details on the tow-level exploratory factor models.

^8^Note that on the SP factor items from all three theoretically postulated factors of centrality (Berntsen and Rubin, 2006) loaded: Item 1 (Identity), Item 2 (Identity), Item 3 (Turning Point), Item 4 (Reference Point). On the LC factor, items from two theoretically postulated factors loaded: Item 5 (Turning Point), Item 6 (Reference Point), Item 7 (Turning Point).

^9^These residual covariances appear justified based on the assumption that there is an individual, idiosyncratic tendency to rate the respective positive and negative CES items similarly, e.g., generally endorsing Item 1 strongly for both positive and negative events.

^10^The residual covariance between Items 1 and 2 is most likely due to both items belonging to the “identity” factor of the full CES. For Items 2 and 4, there is no obvious reason for a residual covariance.

^11^Note that factor correlations (or standardized covariances) are much more similar across levels (see Figure 1), which results from the fact that factor variances were also much larger on Level 1. For a comparison of the strength of relationships among factors across levels, however, covariances represent the more adequate metric because correlations are based on the assumption of equal variances—which obviously does not hold (see Figure 2).

^12^Only for the Self-Perception factor strong invariance was partial (because Item 4 is an indicator of it), while for Life-Course full strong invariance held across event type.

## Appendix

**Table A1:** Factor loadings in multilevel exploratory factor analyses of the brief CES.

	**One-factor model**		**Two-factor model**			
	**Within**	**Between**	**Within**		**Between**	
**Item**	**Factor 1**	**Factor 1**	**Factor 1**	**Factor 2**	**Factor 1**	**Factor 2**
	**Positive events**					
1	0.76	0.88	0.52	0.35	0.95	0.00
2	0.69	0.92	0.89	0.00	0.76	0.26
3	0.86	0.85	0.55	0.38	0.68	0.32
4	0.66	0.93	0.64	0.14	0.54	0.26
5	0.89	0.76	0.00	0.93	0.08	0.89
6	0.69	0.40	0.09	0.62	−0.08	0.77
7	0.84	0.67	0.00	0.87	0.00	0.93
	**Negative events**					
1	0.75	0.89	0.46	0.33	0.89	0.00
2	0.70	0.93	0.88	0.00	0.94	−0.02
3	0.84	0.95	0.53	0.34	0.57	0.32
4	0.67	0.88	0.60	0.18	0.91	0.00
5	0.84	0.91	0.00	0.91	0.00	0.91
6	0.60	0.69	0.12	0.52	0.15	0.62
7	0.79	0.86	0.00	0.83	0.00	0.94

Geomin rotation was used in the two-factor model. Factor loadings were estimated using Maximum Likelihood.
